# Postpartum Delirium: A Psychosis Born in the 18th Century

**DOI:** 10.1192/j.eurpsy.2023.2398

**Published:** 2023-07-19

**Authors:** J. R. Martins, R. Vaz, A. L. Costa, J. Brás, R. Sousa, J. Abreu, E. Almeida, R. Andrade, N. Castro, T. Casanova

**Affiliations:** 1 Centro Hospitalar Tondela-Viseu, Viseu, Portugal

## Abstract

**Introduction:**

Pregnancy and childbirth are moments of great vulnerability in a woman’s life, which can predispose her to the development of psychopathology, ranging from transient depressive symptoms (“baby blues”) to psychotic symptoms. Postpartum delirium is the psychiatric syndrome that some authors refer to as puerperal psychosis par excellence. It was first described in the 18th century and were thought to be associated with painful delivery, then became rare after the introduction of effective analgesia.

**Objectives:**

The objective of this work is to contribute to a better understanding of this condition, through a literature review.

**Methods:**

Bibliographic research using Pubmed® and the keywords: postpartum delirium.

**Results:**

Clinical presentation of postpartum delirium includes: constantly varying degrees of consciousness; perplexity; hallucinations or pseudo-hallucinations of one or more organs of sense; delusions or delusive-type thoughts; great motoric unrest and considerable motoric and verbal abandon; and acute aggressive discharges can also occur. It is thought to be due to organic complications, such as infectious disease, abnormal loss of blood, thrombosis, neurological disease, obstetric disease, vitamin deficiencies, hormonal changes. An article from 1975 mentions how difficult was to treat postpartum delirium despite the development of psychopharmaceutical therapy. The patients remained psychotic for long periods and had many relapses. They mention a comparative study that found that the symptomatic treatment of this syndrome with a combination of perfenazine and lithium carbonate produced relatively favorable results. For that reason, at that time, it was the medication of choice. Nowadays the psychopharmacological treatment of puerperal psychosis, in general, still consists of the combination of lithium and an antipsychotic, such as haloperidol, and possibly a benzodiazepine, such as lorazepam.

**Conclusions:**

Postpartum delirium is rarely mentioned in the literature and just a few cases have been described. It is considered a rare postpartum psychotic condition but would perhaps be less rare if its existence were recognized. On this note, it is important for clinical practice to research on the psychoses of pregnancy and not just the most common.

**Disclosure of Interest:**

None Declared

